# Mitochondrial coding genome analysis of tropical root-knot nematodes (*Meloidogyne*) supports haplotype based diagnostics and reveals evidence of recent reticulate evolution

**DOI:** 10.1038/srep22591

**Published:** 2016-03-04

**Authors:** Toon Janssen, Gerrit Karssen, Myrtle Verhaeven, Danny Coyne, Wim Bert

**Affiliations:** 1 Nematology Research Unit, Department of Biology, Ghent University, K.L. Ledeganckstraat 35, 9000 Ghent, Belgium; 2 National Plant Protection Organization, Wageningen Nematode Collection, P.O. Box 9102, 6700 HC Wageningen, The Netherlands; 3 International Institute of Tropical Agriculture (IITA), c/o icipe, Kasarani, P.O. Box 30772-00100, Nairobi, Kenya

## Abstract

The polyphagous parthenogenetic root-knot nematodes of the genus *Meloidogyne* are considered to be the most significant nematode pest in sub-tropical and tropical agriculture. Despite the crucial need for correct diagnosis, identification of these pathogens remains problematic. The traditionally used diagnostic strategies, including morphometrics, host-range tests, biochemical and molecular techniques, now appear to be unreliable due to the recently-suggested hybrid origin of root-knot nematodes. In order to determine a suitable barcode region for these pathogens nine quickly-evolving mitochondrial coding genes were screened. Resulting haplotype networks revealed closely related lineages indicating a recent speciation, an anthropogenic-aided distribution through agricultural practices, and evidence for reticulate evolution within *M. arenaria*. Nonetheless, nucleotide polymorphisms harbor enough variation to distinguish these closely-related lineages. Furthermore, completeness of lineage sorting was verified by screening 80 populations from widespread geographical origins and variable hosts. Importantly, our results indicate that mitochondrial haplotypes are strongly linked and consistent with traditional esterase isozyme patterns, suggesting that different parthenogenetic lineages can be reliably identified using mitochondrial haplotypes. The study indicates that the barcode region Nad5 can reliably identify the major lineages of tropical root-knot nematodes.

Root-knot nematodes of the genus *Meloidogyne* are considered to be the most significant nematode pest to crop production, causing multi-billion dollar annual losses worldwide^1^. Indeed, one species, *M. incognita*, is considered to be the world’s most damaging crop pathogen^2^. However, despite the crucial need for correct diagnosis of these pathogens, identification of root-knot nematodes continues to pose an obstacle, even for specialists, with reliable, routine identification methods far from established. Traditionally, researchers have relied on morphometrics and perennial patterns for species identification, which is now known to be greatly hampered by phenotypic plasticity and interspecific similarities^3^. Hartman and Sasser^4^ also devised a technique based on differential host preferences, even though, to date, no genetic, isozymatic, or cytogenetic basis has been established for these different host races, indicating that they do not in fact represent homological speciation events^5^. Moreover, increasing the number of host plants has inevitably led to additional races^6^. It has also been suggested that the virulence of *Meloidogyne* is mediated by epigenetic control^7^, thus rendering host specificity an inappropriate diagnostic technique.

In the mid-1980’s Esbenshade and Triantaphyllou^8^,^9^ developed a biochemical-based diagnostic technique, reliant on isozyme profiles; variations in esterase and malate dehydrogenase isozyme profiles proved extremely informative in differentiating most known *Meloidogyne* species. The main drawback to this method, however, is that the technique is only applicable to young adult females, with results often varying between laboratories, leading to suggestions that polymorphic enzyme profiles exist^9^. Despite these shortcomings, isozyme electrophoresis remains one of the most reliable and widely-used differentiation methods^10^,^11^,^12^. Further to this, biochemical techniques involving monoclonal or polyclonal antibodies appeared promising, yet additional research and validations are required before this method can be applied in practice^13^.

The first molecular diagnostic techniques to differentiate *Meloidogyne* species were introduced by Curran *et al*.^14^, using restriction length polymorphisms (RFLP) of genomic DNA. Microsatellites, often in combination with probes, have also been explored for specific diagnosis^15^,^16^. Despite the fact that several of these techniques can distinguish between various *Meloidogyne* species, none is used as frequently as the species-specific primers method. Species-specific primers have been developed to amplify sequence-characterised amplified regions (SCAR), which have been converted from diagnostic randomly amplified polymorphic DNA fragments (RAPDs)^17^,^18^,^19^. This gel based technique is simple, life-stage independent, cost efficient and permits numerous samples to be run within a reasonable amount of time. In addition, the technique is regularly updated as new species-specific primers are developed^20^. However, some challenges remain associated with species-specific primers, such as ambiguous results, low sensitivity, occasionally poor band visibility, and lack of reproducibility between laboratories^12^,^21^.

With the rapidly declining cost and improved availability of genetic sequencing and related technologies, theoretically, all the aforementioned methods could be replaced by DNA barcoding^22^. However, the search for an appropriate barcode region has so far proved notoriously difficult, especially for a clade of mostly mitotic parthenogenetic pathogens including *M. incognita*, *M. javanica*, and *M. arenaria*, commonly referred to as “tropical root-knot nematodes” or the *M. incognita* group (MIG)^23^,^24^. These closely-related MIG lineages, together with the divergent *M. enterolobii*, form a phylogenetically, well-supported group, named clade I^25^,^26^.

The first barcode region to be evaluated was the ribosomal gene cluster but 18S and 28S rDNA appear to lack the resolution required to distinguish between these closely-related lineages^27^. Conversely, the quickly-evolving internal transcribed spacer (ITS) regions have been shown to contain multiple, highly divergent copies within a single individual^28^. These divergent copies could well be linked to the hybrid origin of parthenogenetic root-knot nematodes, as suggested by Lunt *et al*.^29^, which would create difficulties for barcoding these hybrid lineages using nuclear markers^29^,^30^. Arguably, mitochondrial genes can partly circumvent these problems due to their uni-parental inheritance and high mutation rate^24^,^31^. Therefore the intergenic region between 16s and Cox2 has become a focus for characterizing parthenogenetic *Meloidogyne* lineages. Based on this region a PCR-based detection method for root-knot nematodes was developed^32^,^33^ and using root-knot nematode species from Australian restriction fragment analysis of this region revealed a correspondence with isozyme phenotypes^34^. Recently, progress towards a more reliable and durable technique was made by Pagan *et al*.^24^; as several MIG lineages could each be assigned unique mitochondrial haplotypes based on PCR fragment size and restriction cleavage patterns, which was assessed on a range of ethanol-preserved populations from Africa. In search of a suitable barcode region for the MIG, the traditionally-used cytochrome c oxidase 1 and 2 regions are reportedly insufficiently variable to reliably distinguish MIG lineages^35^. Nonetheless, the development of a reliable barcode marker for these root-knot nematodes is of huge economic significance since correct identification of these pathogens can be critical for making informed decisions for efficient and suitable management strategies.

A reliable barcode marker should preferably be a mitochondrial coding region, as intergenic regions have been shown to contain AT repeats that appear not to correlate with speciation events^24^,^36^. The goal of the current study therefore, was to verify whether the coding genes of the mitochondrial genome of clade I root-knot nematodes harbor useful diagnostic barcoding regions. Primers for nine coding genes of the mitochondrial genome were developed. These were sequenced, screened for polymorphic nucleotide positions, and compared with traditional isozyme electrophoresis profiles. To ascertain if lineage sorting of polymorphic positions was complete, numerous populations from geographically widespread origins and variable host plants were screened. The ultimate aim was to provide a simple, efficient and reproducible barcoding assay for reliable identification of MIG pathogens.

## Results

### Sampling and isozyme electrophoresis

**Figure 1:**
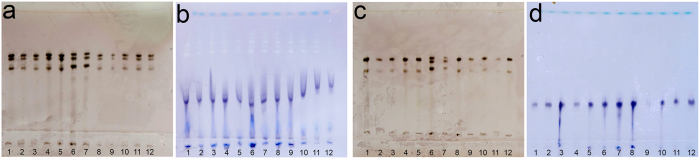
Isozyme profiles of two undescribed MIG lineages. Lane 6 and 7 represent *Meloidogyne javanica* reference phenotypes, lane 1–5 and 8–12 represent undescribed MIG lineages. (**a**) esterase A2-S1-M1 phenotype of *Meloidogyne* sp. 1, (**b**) malate dehydrogenase N1 phenotype of *Meloidogyne* sp. 1, (**c**) esterase A1a-S1 phenotype of *Meloidogyne* sp. 2, (**d**) malate dehydrogenase N1 phenotype of *Meloidogyne* sp. 2.

Among the 80 populations of root-knot nematodes examined 10 different esterase profiles and three different malate dehydrogenase patterns were identified (Table 1). These profiles represent 11 lineages of *Meloidogyne*, of which two appear new to science (see Fig. 1). In total seven populations of *M. enterolobii* were identified, including specimens originating from the type localities of *M. enterolobii* and its junior synonym *M. mayaguensis*^37^. The most frequently-occurring lineages in the dataset were *M. incognita* (28 populations) and *M. javanica* (26 populations), originating from a range of host plants and a wide geographical distribution (Table 1). *Meloidogyne incognita* was represented by both the I1 and I2 phenotype, although the I1 phenotype only occurred when esterase bands were weakly visible, indicating that the absence of the secondary esterase band represents an analysis artifact. For this reason all *M. incognita* esterase phenotypes in this study are defined as I1. *Meloidogyne arenaria* is represented by three isozyme profiles: the A2N1 type (4 populations), the A2N3 type (4 populations) and the A3N1 type (1 population). Three populations of *M. luci*, two populations of *M. inornata* and one population of *M. ethiopica* had an L3, I3 and E3 esterase phenotype, respectively. In addition to these known isozyme patterns two previously unrecorded esterase profiles were discovered (Fig. 1); one occurring in two populations, one originating from China from *Ficus* and one from Tanzania from *Heliconia*. The Mdh pattern of these populations was characterised as the N1 phenotype (Fig. 1b). The esterase phenotype displays three clear bands, of which the two fast migrating bands are positioned in the same location as for the *M. arenaria* A2 phenotype, while the slowest migrating band and its slightly faster migrating weak band occur in a similar position as the S1-M1 phenotype (Fig. 1a). Both the A2 and S1-M1 phenotype have previously been associated with *M. arenaria*^9^ indicating that our combined A2-S1-M1 pattern should be considered an atypical, possibly hybrid, *M. arenaria* pattern. A second novel pattern was associated with two *Meloidogyne* populations, one originating from Spain (Beet) and one from Guatemala (Tomato). The Mdh activity displayed a N1 phenotype (Fig. 1d) and the esterase phenotype consists of two bands of which the faster migrating band is more pronounced (Fig. 1c). This esterase phenotype is not directly related to any other described *Meloidogyne* esterase phenotype indicating a new, undescribed lineage. The slow migrating band is in the S1 position^9^ while the fast migrating band is in a new position, herein named A1a. This new esterase phenotype is therefore referred to as A1a-S1.

### Identification of polymorphic sites within the mitochondrial genome

From the 80 geographically widespread populations, 305 sequences and 22 mitochondrial haplotypes were generated (Table S3), corresponding to 11 isozyme based lineages of clade I root-knot nematodes. Identical results with different primer combinations and different DNA polymerases. Sequence results for multiple individuals within one population (tested for 11 populations, see Table S3). Cloning data revealed limited heteroplasmy within a single individual, but never associated with informative species specific nucleotide positions, *i.e*. 0.16% variation (1 nucleotide position) in one clone of T417, 0.16% variation in two clones of T520, 0.33% variation in one clone of T540, while no variation was detected in T515.

### Meloidogyne enterolobii

The seven populations of *M. enterolobii* showed identical sequences for the eight analysed gene fragments (Table S3), except for the population originating from the type locality of the former *M. mayaguensis*, which displayed a single mutation in the Cox3 fragment. The *M. enterolobii* haplotype was clearly divergent from the MIG lineages, as our gene fragments were different in 29 nucleotide positions (7.7%) in 16S, 93 positions (11.1%) in Cox1, 30 positions (7.5%) in Cox2, 39 positions (10.8%) in Cox3, 81 positions (10.8%) in Cytb, 68 positions (15.4%) in Nad1, 22 positions (10.1%) in Nad3 and 79 positions (18%) in Nad5, placing *M. enterolobii* in a clearly phylogenetically distinct position within clade I.

### MIG lineages

The 16S fragment revealed six polymorphic sites (1.5%), including two *M. javanica*-specific mutations, a *M. incognita* and a *Meloidogyne* sp. 2-specific site (Fig. S1). Additionally, in one population of *M. arenaria* and one population of *M. incognita* an additional single mutation within the 16S fragment was encountered.

The Cox1 fragment contained seven variable positions, but with only 0.7% variable sites and five different haplotypes, this gene is one of the most conserved regions sequenced in this study (Fig. S2). Nevertheless, it revealed five *Meloidogyne* sp. 2-specific sites, one *Meloidogyne* sp. 1-specific mutation and two sites displaying variability between different populations of *M. luci* and *M. inornata*.

Our Cox2 fragment revealed five haplotypes (Fig. S1). One is shared between *M. luci* and *M. inornata*, and one is shared among 10 populations of *M. incognita* and our only representative population of *M. ethiopica*. A third haplotype has two *Meloidogyne* sp. 2-specific sites. Cox2 was not able to differentiate between *M. arenaria*, *M. javanica*, *M. floridensis* and *Meloidogyne* sp. 1 H1, which are grouped in a fourth haplotype, while a fifth haplotype composes *Meloidogyne* sp. 1 H2.

The Cox3 gene fragment reveals four haplotypes (Fig. S1). One is characteristic for five *M. arenaria* populations including three isozyme types (A2N1, A3N1 and A2N3). A second haplotype has two lineage-specific sites for *Meloidogyne* sp. 2. A third is shared among *M. luci*, *M. ethiopica* and *M. inornata* and a fourth groups *M. javanica*, *M. incognita*, *Meloidogyne* sp. 1, two *M. arenaria* populations and *M. floridensis*.

The Cytb fragment contained a lineage-specific haplotype for *M. ethiopica*, *M. floridensis*, *Meloidogyne* sp. 2 and distinguished several populations of *M. arenaria*, but failed to separate the other lineages included in the current study (Fig. S2).

Our Nad1 gene reveals seven haplotypes (Fig. S2). These represent a *M*. *javanica*, *M. luci*, *M. floridensis*, *Meloidogyne* sp. 1 H1, *Meloidogyne* sp. 2. and an *M. incognita*-specific haplotype, each differing in one nucleotide position. Nad1 did not differentiate between *M. arenaria*, *Meloidogyne* sp. 1 H2, and *M. ethiopica*, although one nucleotide position showed variability between various *M. arenaria* populations.

The Nad2 fragment contains eight polymorphic positions, revealing 10 haplotypes (Fig. S2). A first haplotype is *M. javanica*-specific differing in at least two mutations from the other MIG lineages. Also *Meloidogyne* sp. 2 and *M. inornata* each have a lineage-specific haplotype, while *M. incognita* is represented by four closely-related specific haplotypes. Another haplotype is shared by *M. luci, M. ethiopica* and *M. floridensis*. The final haplotype groups *M. arenaria*, *Meloidogyne* sp. 1 and one population of *M. arenaria* differing in a single nucleotide position.

**Figure 2:**
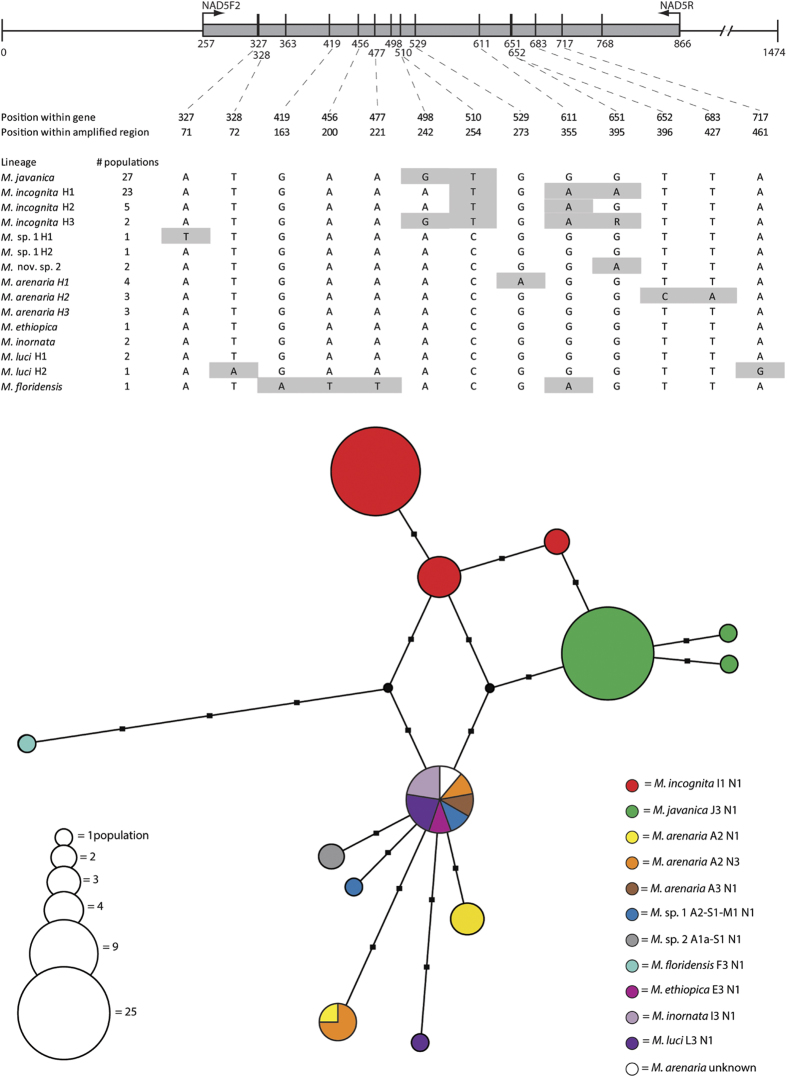
Nad5 barcode gene sequence comparison between MIG lineages. A schematic overview of the gene shows the position and length of the amplified fragment, primer position and position of polymorphic nucleotide positions. Alongside the schematic overview an overview table shows the polymorphic nucleotide positions for comparison with barcode sequences as well as the number of populations studied. The haplotype network shows the relationships between different haplotypes, circle size is equivalent to the number of studied populations and branch length is equivalent to the number of mutations (shown as black squares). Different isozyme phenotypes are displayed by different colours, median vectors are shown as black circles. Within the Nad5 gene two *Meloidogyne javanica* populations (T347 and T417) each have an extra mutation which are not shown in the schematic overview. H1, H2 and H3 indicate different haplotypes of a certain lineage. *Meloidogyne incognita* H3 displays a heterozygous position at site 395 indicated with a degenerate base “R” in the table.

The Nad3 fragment was identical for all 17 representative sequenced populations (Table S3). Conversely, with 15 polymorphic positions, the Nad5 fragment is the most variable with a variation of 2.46% and representing 13 haplotypes out of 78 sequenced populations (Fig. 2). The *M. incognita* I1 esterase type is represented by three closely-related haplotypes differing in only one or two positions from each other. Of 27 *M. javanica* populations, 25 shared the same haplotype, while two populations had closely-related haplotypes differing in only one position. Another common haplotype was shared by *M. inornata*, *Meloidogyne* sp. 1, *M. arenaria* H3, *M. ethiopica* and two of the three *M. luci* populations. This haplotype most likely corresponds to the haplotype G, as defined and recovered by Pagan *et al*.^24^ based on restriction fragment analysis of the intergenic region between 16S and Cox2. The third *M. luci* population included in the current study had a slightly different haplotype, differing in two positions from the other two populations and also *Meloidogyne* sp. 1, which is associated with a second distinct haplotype. Additionally, two *M. arenaria* haplotypes were recovered, each differing in one or two nucleotide positions. However, no link was observed between isozyme phenotypes and mitochondrial haplotypes among the different populations of *M. arenaria*. *Meloidogyne floridensis* is associated with the most divergent haplotype, differing in at least four positions to its closest relatives. Finally for the Nad5 fragment, a lineage-specific haplotype was recorded for *Meloidogyne* sp. 2.

### Multi-gene haplotype network

**Figure 3:**
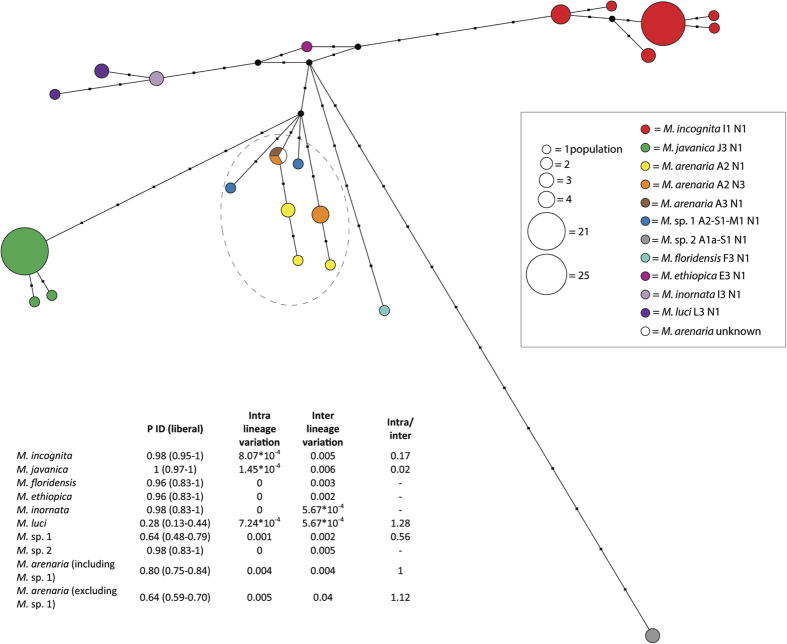
Multi-gene haplotype network of studied MIG lineages as calculated from 16S, Cox1, Cox2, Cox3, Nad2 and Nad5 gene fragments. The haplotype network shows the relationships between different haplotypes, circle size is equivalent to the number of studied populations and branch length is equivalent to the number of mutations (shown as black squares). Different isozyme phenotypes are displayed by different colours, median vectors are shown as black circles. The *Meloidogyne arenaria* group is highlighted by a dashed circle. The table shows P ID (liberal) values indicating the probability of correctly identifying these lineages using BLAST, DNA Barcoding or tree placement; intra-lineage variation; inter-lineage variation to closest neighboring lineage and a ratio of intra- and inter lineage specific variation indicating the degree of separation of the lineage.

A haplotype network was constructed using a concatenated alignment of the Cox1, Cox2, Cox3, 16S, Nad2 and Nad5 gene fragments. This multi-gene haplotype network clearly separates the major lineages of root-knot nematodes (Fig. 3). Both *M. javanica*, *M. incognita*, *M. floridensis* and *Meloidogyne* sp. 2 each occur in a clearly-separated position, supported by several lineage-specific sites. This separated position is confirmed by the low intra-/inter-lineage variability ratio of these lineages (Fig. 3). This intra-/inter variability ratio is higher for *M. arenaria* and *M. luci*, suggesting that intra-lineage variability is lower relative to inter-lineage variability with the nearest neighboring lineage. High P ID (liberal) values^38^ indicate a high probability of correctly identifying these lineages using BLAST, DNA Barcoding or tree placement. Interestingly, however, both *M. incognita* and *M. javanica* show some intraspecific mitochondrial variability. Furthermore, the closely related isozyme phenotypes of *M. ethiopica*, *M. inornata* and *M. luci* occupy separate positions in accordance to their mitochondrial haplotypes. Two distinct haplotypes for *M. luci* were also observed, occurring in a paraphyletic position, one shared between two populations originating from Iran and Slovenia and another haplotype from Guatemala. All ten included *M. arenaria* populations form a largely unresolved cloud of closely related haplotypes (Fig. 3). Surprisingly, the A3 esterase phenotype shared a haplotype with an A2 phenotype population and the haplotype extracted from the mitochondrial genome of *M. arenaria*^39^ for which the isozyme profile is unknown. This indicates that different isozyme phenotypes are not necessarily associated with different mitochondrial haplotypes. The slightly different haplotypes of the two *Meloidogyne* sp. 1 populations are closely associated with the *M. arenaria* cloud, indicating that *Meloidogyne* sp. 1 should be considered an *M. arenaria* variant, as already indicated by its esterase isozyme phenotype. Overall, the concatenated mitochondrial haplotype network clearly separates different lineages of parthenogenetic root-knot nematodes, demonstrating a clear link with isozyme phenotypes.

## Discussion

Although the appointment of lineage-specific barcodes for MIG root-knot nematodes is known to be problematic, since several nuclear and mitochondrial candidate genes were found to be unsuitable^28^,^30^,^35^, we were able to find consistent differences between 11 isozyme lineages of root-knot nematodes based on nucleotide polymorphisms originating from nine coding genes of the mitochondrial genome. While non-coding genes have been shown to contain insertions prone to heteroplasmy^24^,^36^,^39^,^40^, we found no evidence for variable insertions within coding genes, only a very limited amount of heteroplasmic positions within a single individual were recovered. However, this variation was not associated with species specific nucleotide positions, indicating that barcode accuracy is not influenced by heteroplasmy.

As previously highlighted in various studies^30^,^35^,^39^,^41^ the only clearly divergent species in clade I is *M. enterolobii*, differing in all seven sequenced mitochondrial gene fragments (7.5%–18% divergent). Consequently *M. enterolobii* can easily be identified using any of the sequenced mitochondrial coding gene fragments. Moreover, its haplotype is identical to the mitochondrial genome sequence of *M. enterolobii*^39^ with virtually no mitochondrial variation between the seven geographically widespread populations of *M. enterolobii* observed. Between the type locality of *M. mayaguensis*^42^ and *M. enterolobii*^43^, just one single mutation in Cox3 was observed. However, this single mutation is considered insufficient to re-erect *M. mayaguensis* as a separate taxon, and thus further supports the synonymisation between *M. mayaguensis* and *M. enterolobii*^37^ based on host range, isozyme phenotype and morphological data.

Except for *M. enterolobii*, clade I comprises extremely closely-related parthenogenetic lineages, known as the MIG^30^,^34^,^39^,^41^,^44^. This close relationship is here confirmed, based on the mitochondrial coding genes, such as the Nad3 gene fragment, which is completely identical for all MIG lineages. Also, the widely used barcode gene Cox1 is too conserved^35^, as it can only reliably differentiate *Meloidogyne* sp. 2 from the other MIG lineages. The limited diversity of mitochondrial coding genes confirms the recent origin of these MIG root-knot nematodes^30^, yet our study reveals that most of the mitochondrial coding genes exhibit some degree of diversity, generally varying between 0 and 1.5%, resulting in informative mitochondrial haplotypes. Remarkably, these mitochondrial haplotypes correspond clearly with isozyme patterns. This reflects earlier restriction fragment analysis of the intergenic region between 16S and Cox2^34^, indicating that the low, but consistent, diversity between different haplotypes can be informative in lineage identification.

Pagan *et al*.^24^ described one *M. incognita*-specific and one *M. javanica*-specific site within the 16S gene, which were subsequently used as lineage-specific restriction sites, the specificity of which was confirmed using numerous root-knot nematode populations from Africa. In the current study, we further confirm these lineage-specific sites, and additionally identify four more *M. incognita*-specific sites and five *M. javanica*-specific sites, which are directly connected to I1 (I2) and J3 esterase phenotypes, respectively. The specificity of these sites was confirmed based on 30 *M. incognita* and 27 *M. javanica* populations of widespread geographic origin, indicating that lineage sorting is complete. Moreover, the most common *M. incognita* mitochondrial haplotype was identical to the haplotype from the mitochondrial genome sequence of *M. incognita* generated by Humphreys-Pereira & Elling^45^ and to the mitochondrial haplotype extracted from the complete genome sequence of *M. incognita*^46^. Also, the most common *M. javanica* haplotype corresponded with its recently published mitochondrial genome^39^. We also found that the unique three banded *M. floridensis* esterase phenotype is associated with a lineage-specific mitochondrial haplotype containing seven lineage-specific sites and a separate position in the haplotype network. This confers with its aberrant meiotic parthenogenetic mode of reproduction and its isolated position according to RAPD data^47^.

Furthermore, our analysis indicates that even very closely-related esterase phenotypes can be reliably identified using mitochondrial haplotypes. For example, the E3, I3 and L3 phenotypes of *M. ethiopica*, *M. inornata* and *M. luci*, respectively, were for the first time connected to distinct but related haplotypes. Surprisingly though, we recovered two separate haplotypes for *M. luci*, which were both more closely related to *M. inornata* than to the other *M. luci* haplotype. Both haplotypes occurred in populations originating from separate geographical regions (e.g. Guatemala, Iran and Slovenia), indicating either that the L3 phenotype evolved convergently from the I3 pattern, or alternatively, that the I3 phenotype is the result of reticulate evolution, which was recently suggested to play an important role during the evolution of the MIG^29^. This latter scenario seems plausible as the E3, I3 and L3 phenotypes are associated with striking variations in somatic chromosome numbers, varying from 2n = 36–38 over 2n = 42–46 to 3n = 54–58, respectively^48^, inferring that these haplotypes and associated isozyme profiles originate from different hybridization events. To clarify the precise origin of these closely-related parthenogenic lineages and their taxonomic status, additional assessment of a broader range of populations from across a wide geographic distribution, in combination with genomic analysis, is necessary.

The three isozyme patterns observed for *Meloidogyne arenaria* (A2N1, A3N1, A2N3) are represented by a largely-unresolved cloud of related haplotypes in our network, where some level of variability is observed. Specifically the A2N3 phenotype occurs in two separate positions in the network, some displaying an identical mitochondrial haplotype with an A3N1 phenotype population, while the A2N1 phenotype appears to be linked to different mitochondrial haplotypes, indicating that Mdh phenotypes are not lineage-specific. Interestingly, intraspecific variability of both H1 and H3 Mdh phenotypes, has already been reported for *M. mali*^49^. That different isozyme phenotypes can be associated with the same mitochondrial haplotype in *M. arenaria* is consistent with a wide variation in karyology, with chromosome numbers varying from 30–38 over 40–48 to 51–56^9^,^50^, indicating several levels of polyploidy. Consequently, the available evidence combined indicates that different lineages of *M. arenaria* have been involved in recent hybridization events. This assumption is further supported by the fact that the A3 phenotype appears to be associated with higher (52–54) chromosome numbers^9^, while sharing all its esterase alleles with the A1 phenotype (absent in our analysis) and the A2 phenotype. Moreover, the A2 S1-M1 phenotype of *Meloidogyne* sp. 1 (haplotypes are close to the *M. arenaria* cloud) appears to be a combination of two previously reported *M. arenaria* phenotypes (A2 and S1-M1)^9^. This suggests that *M. arenaria* actually comprises a random combination of lineages with different isozyme phenotypes and mitochondrial haplotypes.

The newly described esterase isozyme phenotype (Fig. 1c,d) of *Meloidogyne* sp. 2 shows a very distinct mitochondrial haplotype, displaying 16 lineage-specific mutations, establishing it as the most divergent lineage of the MIG to date (Fig. 3). Additional information on this deviating lineage, which appears to have a wide geographical distribution (Spain and Guatemala) is necessary, including its mode of reproduction, cytogenetic composition and host range, in order to understand its divergent phylogenetic position. The observation that the two newly determined isozyme patterns also relate to distinct mitochondrial haplotypes provides a strong indication of the high potential value of mitochondrial haplotypes for separating lineages and root-knot nematode diagnostics. With the exception of *M. arenaria* A3, it is further demonstrated that each esterase phenotype is associated with a specific mitochondrial haplotype and that our multi-gene haplotype network shows a high degree of resemblance with the phylogenetic tree of Esbenshade and Triantaphyllou^8^ as derived from the evaluation of isozymic data. This accordance between biochemical and molecular identification techniques provides a great opportunity to evaluate *Meloidogyne* species concepts, especially in combination with upcoming genomic information.

Due to the mostly parthenogenetic nature and suggested hybrid origin of *Meloidogyne* lineages in clade I (Lunt *et al*. 2014), it is difficult to link mitochondrial haplotypes with actual speciation events. Haplotype variation can occur among lineages with the same isozymic pattern (e.g. *Meloidogyne* sp. 1, *M. Luci, M. incognita, M. javanica*), while in rare cases, reticulate evolution enables species to possess the same mitochondrial haplotype but different isozymatic patterns, indicating a different genomic composition (e.g. *M. arenaria* A3 and A2, see above). Haplotype variation may be a consequence of accumulated mutations following hybridization and can be considered intraspecific variability. Alternatively, these nucleotide polymorphisms could reflect the diversity generated by crosses within an ancestral gene pool, which were later fixed by hybridization and parthenogenesis^41^. In the latter case, individual mitochondrial haplotypes can be considered separate hybrid lineages, possibly each with a distinct genomic composition. Arguably, both explanations may have played a role in shaping the presently-observed genetic diversity within root-knot nematode mitochondrial genomes. To unravel the precise origin and diversity of clade I mitochondrial haplotypes, additional knowledge on the structure and origin of their genome is crucial towards revealing whether hybridization in this group is traceable to unique hybridization events or, alternatively, that hybridization is rampant and constantly leads to new lineages of parthenogenetic root-knot nematodes. Nevertheless, in both scenarios a mitochondrial haplotype-based identification is preferable over nuclear gene-based identification or morphological determination^5^,^24^,^34^, especially since mitochondrial haplotypes are unequivocally linked with isozyme phenotypes, which continue to be considered a superior diagnostic strategy for root-knot nematodes^11^,^12^. Furthermore, it is suggested that while the species conundrum within the MIG continues to be resolved, ‘lineages’ should be used as a preferred term over ‘species’. Yet, for convenience, the term species remains useful for the well-established ‘species’, although in effect they represent a more or less random combination of lineages.

Interestingly, most root-knot nematode lineages identified in the current study have a global distribution (Table 1). The observation that identical multi-gene mitochondrial haplotypes can have a global distribution favors the hypothesis that this distribution was caused by humans through agricultural practices and does not pre-date human crop exchange and agricultural development^5^. If worldwide distribution would pre-date agricultural development a much larger variation in mitochondrial haplotypes between lineages from distant locations could be expected, especially because parthenogenetic reproduction most likely implies that single nucleotide polymorphisms remain separated between different populations.

The current study demonstrates that root-knot nematodes from clade I can be reliably identified using mitochondrial haplotypes. The Nad5 gene fragment contains the largest number of variable positions and is therefore the preferred barcoding gene for clade I *Meloidogyne* spp. Sequencing the Nad5 fragment allows a reliable identification of the most common MIG lineages, i.e. *M. incognita*, *M. javanica*, most populations of *M. arenaria* but also *M. floridensis and Meloidogyne* sp. 2. However, the relatively uncommon, closely-related lineages i.e. *M. ethiopica*, *M. inornata, M. luci, Meloidogyne* sp. 1 and some *M. arenaria* are clustered in one Nad5 haplotype. In comparison, most of these lineages were also grouped in the same haplotype by Pagan *et al*.^24^ (therein described as haplotype G) based on restriction fragment analysis of the intergenic spacer between Cox2 and 16S. To separate these closely-related lineages requires sequencing of an additional gene (Cox2), preferably in combination with isozyme electrophoresis. In comparison with other diagnostic strategies the proposed DNA barcoding method has several distinct advantages: (i) it is not life stage dependent, which is vital in studying root-knot nematodes, as second-stage juveniles (and males) represent the only free-living stage^5^,^19^; (ii) a single individual is sufficient, which is important as species mixtures are common; (iii) barcoding does not provide a yes/no answer but does help to identify unforeseen plant threats or unknown lineages; (iv) the protocol can be performed in a relatively short time span, in combination with the suggested quick DNA extraction method, which omits a time-consuming proteinase K step, enabling sequence-based lineage identification within a single day; (v) the resulting sequences can be analysed in a comparative population genetic framework using haplotype networks; (vi) barcoding using coding genes does not suffer from heteroplasmy between or within single individuals; (vii) and possibly most importantly, barcoding can produce highly reproducible results between laboratories.

## Methods

### Collection of populations, morphological identification and culturing

For this study 85 separate *Meloidogyne* populations were examined. Thirty-seven populations were obtained from pure cultures originating from the National Plant Protection Organization (Wageningen, the Netherlands), while the other populations were collected during four field surveys in three countries. Sampling of field-cultivated crops was undertaken in Tanzania, Pakistan, and Nigeria between 2012 and 2013, providing 48 populations. Comprehensive information on the geographical origin and the host plant species was collected for all populations (Table 1), which were all morphologically characterised based on second-stage juveniles^51^ and perennial patterns, when females were available, in order to ensure clade I species were included. Subsequently, each population was inoculated onto *Lycopersicon esculentum* cv. Moneymaker plants, individually, in pots containing sterile potting media, using a few egg masses or juveniles. Populations were maintained in the greenhouse at Wageningen at 23 °C.

### Isozyme analysis

To confirm the morphological identification and purity of the cultures, esterase and malate dehydrogenase isozymes were analysed according to Karssen *et al*.^52^. First, ten young females of each culture were isolated from roots in isotonic (0.9%) NaCl solution. Individual females, after desalting in reagent-grade water on ice for 5 minutes, were loaded to sample wells containing 0.6 μl extraction buffer (20% sucrose, 2% triton X-100, 0.01% Bromophenol Blue), and subsequently macerated using a glass rod. This mixture was homogenised, and protein extractions were loaded onto a (8–25) polyacrylamide gradient gel and electrophoretically fractioned using a PhastSystem (Pharmacia Ltd, Uppsala, Sweden). In addition to the ten test samples, two *M. javanica* protein extractions were added to the centre of each gel to serve as a reference. After electrophoresis, gels were stained to examine for malate dehydrogenase (Mdh) and esterase (Est) activity for 5 and 45 minutes, respectively, rinsed with distilled water, and fixed using a 10% glycerol, 10% acetic acid, distilled water solution.

### Mitochondrial DNA analysis

Genomic DNA of crushed individual females was extracted using worm lysis buffer and proteinase K^53^. Genomic DNA of individual second-stage juveniles or males was extracted using a quick alkaline lysis protocol adapted from Schneider *et al*.^54^; individual nematodes were transferred to 10 μl 0.05N NaOH, with 1 μl of 4.5% tween added. The mixture was heated to 95 °C for 15 min, and after cooling to room temperature 40 μl of double-distilled water was added.

PCR primers were designed for 16S ribosomal RNA (16S), the Cytochrome c oxidase subunits 1, 2 and 3 (Cox1, Cox2, Cox3), Cytochrome b (Cytb), the NADH dehydrogenase subunits 1, 2, 3 and 5 (Nad1, Nad2, Nad3, Nad5) using PRIMER3^55^ implemented in GENEIOUS R6 (Biomatters; http://www.geneious.com). As a starting point for primer design a combination of recently published mitochondrial genomes: *M. incognita*, *M. chitwoodi*^45^, *M. graminicola*^56^ and contigs from the genomic next generation sequence data of *M. incognita*^46^, *M. hapla*^57^ and *M. floridensis*^29^ sequencing consortia were used. The resulting primer sequences are displayed in Table 2 together with the length, position and proportion of the amplified fragments.

PCR amplification was carried out using the standard Taq DNA polymerase mixture (Qiagen, Germany), employing 2 μl genomic DNA extraction and 0.4 mM of each primer. The PCR amplification conditions were: initial desaturation for 2 min at 94 °C, followed by 40 cycles of 60 secs at 94 °C, 60 secs at 45 °C, 90 secs at 72 °C, and finally an extension for 10 min at 72 °C. For NADH dehydrogenase subunit 1, Cytochrome c oxidase and Cytochrome b the annealing temperature was increased to 55 °C. PCR products were electrophoretically fractioned on a 1% agarose gel and stained with ethidium bromide. Successful reactions were purified and sequenced commercially by Macrogen Inc. (Europe) in forward and reverse direction. Consensus sequences were assembled using GENEIOUS R6. All contigs were subjected to a BLAST search on the NCBI website (http://www.ncbi.nlm.nih.gov) to check for possible contaminations. Reliability and reproducibility of PCR amplification was tested by sequencing Nad1 twice using a different primer combination NAD1F1 (TCA AAT TCG TTT AGG ACC AAC) and Nad1R1 (CGA ATT GTT TAT CCT CGT TTT C) and by substituting Taq DNA polymerase by Phusion^®^ High-Fidelity DNA Polymerase. To check heteroplasmy within a population and within a single individual, respectively multiple individuals of a single population were sequenced and four populations were cloned using pGEM^®^-T Easy Vector Systems Promega *i.e*. *M. javanica* T417 (3 sequenced clones), *M. javanica* T520 (9 sequenced clones), *M. incognita* T515 (3 sequenced clones) and *M. incognita* T540 (4 sequenced clones). All mitochondrial sequences were translated on the TranslatorX web server^58^ using the invertebrate genetic code and aligned by its amino acid translation using MAFFT 7.157^59^. Haplotype networks were calculated using the median joining algorithm as implemented in Network 4.6^60^, http://www.fluxus-engineering.com/), gaps were coded as unknown characters. Haplotype diagrams were redrawn in ADOBE^®^ ILLUSTRATOR^®^ CS3. Liberal P ID values, inter- and intra-lineage species variability were calculated with the species delineation plugin of GENEIOUS R6^38^ using a UPGMA tree, and distances were calculated according to the Jukes-Cantor model. In all analyses the generated sequences in the current study were complemented with mitochondrial haplotypes extracted from the mitochondrial genome sequences of *M. incognita*^45^, *M. javanica*, *M. enterolobii* and *M. arenaria*^39^ and haplotypes extracted from whole genome sequences of *M. incognita*^46^ and *M. floridensis*^29^.

## Additional Information

**How to cite this article**: Janssen, T. *et al*. Mitochondrial coding genome analysis of tropical root-knot nematodes (*Meloidogyne*) supports haplotype based diagnostics and reveals evidence of recent reticulate evolution. *Sci. Rep*. **6**, 22591; doi: 10.1038/srep22591 (2016).

## Supplementary Material

Supplementary Information

## Figures and Tables

**Table 1:** Studied populations with their unique ID number together with esterase isozyme phenotype (Est), malate dehydrogenase isozyme phenotype (Mdh) and their respective host plant.

Species	Specimen ID	Est	Mdh	Host plant	Location	GPS coordinates/reference culture number
***Meloidogyne enterolobii***						
	T337	VS1-S1	N1a	*Solanum melongena*	Puerto rico, typematerial *Meloidogyne mayaguensis* received from Dr. V. Blok	E1834
	T382	VS1-S1	N1a	*Enterolobium contortisiliquum*	China, Hainan Island, type locality sample	E1470
	T424	VS1-S1	N1a	*Solanum lycopersicum*	USA, Florida, received from Dr. J. Brito	E7314-1 (no1-514-313 5/15/04)
	T441	VS1-S1	N1a	Cactaceae	Singapore, received from Dr. A. Castillo	E8336
	T463	VS1-S1	N1a	*Ulmus*	China	E4775-1
	T468	VS1-S1	N1a	*Capsicum annuum*	Mexico	E9491
	T536	VS1-S1	N1a	*Chlorophytum*	Fera, R. Lawson 3122513	E9578
***Meloidogyne incognita***						
	T384	I1	N1	*Daucus carota*	Morocco	E5942
	T161	I1	N1	*Ficus*	China	F0983
	T515	I1	N1	*Solanum tuberosum*	Italy	E1318
	T526	I1	N1	*Syngonium*	Togo	R91/2342
	T532	I1	N1	*Vitis*	Egypt, Monufia Governorate, El Sadat city	E9619-30a
	T540	I1	N1	*Philodendron selloum*	United States of America	F1763
	T552	I1	N1	*Ficus*	China	E2107-1
	Y29	I1	N1	*Dioscorea* (Yam)	Nigeria, Kogi, Idah	7°6′56″N, 6°44′37″E
	Y57	I1	N1	*Celosia*	Nigeria, Oyo, Akobo	7°25′57″N, 3°56′35″E
	C33	I1	N1	*Solanum aethiopicum*	Tanzania, Morogoro, Kipera	6°55′56.784″S, 37°32′3.408″E
	C41	I1	N1	*Solanum lycopersicum*	Tanzania, Morogoro, Kipera	6°56′13.452″S, 37°31′35.543″E
	C49	I1	N1	*Solanum aethiopicum*	Tanzania, Morogoro, Mlali	6°58′0.48″S, 37°31′18.12″E
	C53	I1	N1	*Solanum lycopersicum*	Tanzania, Morogoro, Mlali	6°58′0.192″S, 37°31′18.048″E
	C69	I1	N1	*Solanum lycopersicum*	Tanzania, Morogoro, Hembeti	6°17′27.096″S, 37°28′18156″E
	C81	I1	N1	*Solanum lycopersicum*	Tanzania, Morogoro, Msongozi	7°3′59.508″S, 37°20′41.279″E
	C87	I1	N1	*Solanum lycopersicum*	Tanzania, Morogoro, Msongozi	7°3′50.4″S, 37°20′34.044″E
	C95	I1	N1	*Solanum lycopersicum*	Tanzania, Morogoro, Msongozi	7°3′21.024″S, 37°19′53.867″E
	M4	I1	N1	*Capsicum annuum*	Tanzania, Morogoro, Mlali	6°57′4.248″S, 37°31′45.12″E
	M8	I1	N1	*Solanum lycopersicum*	Tanzania, Morogoro, Mlali	6°57′4.248″S, 37°31′45.12″E
	M15	I1	N1	*Capsicum annuum*	Tanzania, Morogoro, Mlumbilo-Mtibwa	6°11′42.72″S, 37°42′54.647″E
	M20	I1	N1	*Fabaceae* (Bean)	Tanzania, Morogoro, Muomero	6°17′52.836″S, 37°26′30.66″E
	M21	I1	N1	*Solanum aethiopicum*	Tanzania, Morogoro, Muomero	6°17′52.836″S, 37°26′30.66″E
	M28	I1	N1	*Solanum lycopersicum*	Tanzania, Pwani, Bagamoyo-mtoni	6°27′10.62″S, 38°53′23.784″E
	M44	I1	N1	*Coffea*	Tanzania, Morogoro, Luale	7°7′57.108″S, 37°32′17.916″E
	M46	I1	N1	*Pisum sativum*	Tanzania, Morogoro, Luale	7°7′58.404″S, 37°32′6.792″E
	M49	I1	N1	*Solanum lycopersicum*	Tanzania, Morogoro, Bunduki	7°1′53.184″S, 37°37′2.891″E
	A1	I1	N1	*Abelmoschus esculentus*	Pakistan, Faisalabad, Chak # 61 JB Dharoran	31°26′53.02″N, 72°58′14.00″E
	A3	I1	N1	*Abelmoschus esculentus*	Pakistan, Faisalabad, Chak # 146/RB II Khewa	31°36′6.34″N, 73°16′39.41″E
***Meloidogyne javanica***						
	T347	J3	N1	*Solanum lycopersicum*	Rwanda, Kayonza	1°57′16.6″S, 30°31′16.9″E
	T417	J3	N1	*Carmona*	China	E9455
	T429	J3	N1	*Solanum lycopersicum*	Spain	F1836-3
	T485	J3	N1	*Ficus*	China	E1090-4
	T497	J3	N1	*Fabaceae* (Bean)	Morocco	E9492
	T509	J3	N1	*Solanum tuberosum*	Congo	E1387
	T520	J3	N1	Pistache	Iran	D4872
	Y60	J3	N1	*Dioscorea* (Yam)	Nigeria, Benue, Tsiabi	7°15′52″N, 8°15′3″E
	C35	J3	N1	*Solanum lycopersicum*	Tanzania, Morogoro, Kipera	6°55′56.784″S, 37°32′3.408″E
	C47	J3	N1	*Solanum lycopersicum*	Tanzania, Morogoro, Mlali	6°57′3.708″S, 37°31′48.37″E
	C63	J3	N1	*Solanum lycopersicum*	Tanzania, Morogoro, Dakawa	6°26′58.236″S, 37°31′53.184″E
	C89	J3	N1	*Solanum lycopersicum*	Tanzania, Morogoro, Msongozi	7°3′14.796″S, 37°22′39.971″E
	M14	J3	N1	*Brassica*	Tanzania, Morogoro, Mlumbilo-Mtibwa	6°11′40.884″S, 37°42′51.552″E
	M30	J3	N1	*Abelmoschus esculentus*	Tanzania, Pwani, Bagamoyo-mtoni	6°27′10.62″S, 38°53′23.784″E
	M39	J3	N1	*Solanum lycopersicum*	Tanzania, Dar-es-Salaam, Kisse	7°0′0″S, 39°0′0″E
	M40	J3	N1	*Brassica oleracea*	Tanzania, Morogoro, Msufini	6°17′15.432″S, 37°28′38.675″E
	M50	J3	N1	*Coffea*	Tanzania, Morogoro, Bunduki	7°1′54.12″S, 37°36′51.804″E
	A8	J3	N1	*Solanum melongena*	Plant Pathology Research Area (Culture), University of Agriculture, Faisalabad	/
	A21	J3	N1	*Abelmoschus esculentus*	Pakistan, Mandibahauddin, Phalia, Kadhar	32°25′48.27″N, 73°28′40.63″E
	A23	J3	N1	*Abelmoschus esculentus*	Pakistan, Mandibahauddin, Phalia, Chhohranwala	32°31′42.40″N, 73°44′4.20″E
	A24	J3	N1	*Abelmoschus esculentus*	Pakistan, Faisalabad, Chak # 225 RB Malkhanwala	31°21′48.23″N, 73° 7′5.87″E
	A25	J3	N1	*Cucurbita pepo*	Pakistan, Mandibahauddin, Phalia, Chhohranwala	32°31′53.60″N, 73°43′23.00″E
	A29	J3	N1	*Solanum melongena*	Pakistan, Mandibahauddin, Phalia, Seeray	32°23′56.21″N, 73°32′35.66″E
	A30	J3	N1	*Cucurbita pepo*	Pakistan, Mandibahauddin, Phalia, Seeray	32°23′56.41″N, 73°32′33.48″E
	A31	J3	N1	*Abelmoschus esculentus*	Pakistan, Mandibahauddin, Phalia, Seeray	32°23′57.95″N, 73°32′35.54″E
	A32	J3	N1	*Cucurbita pepo*	Pakistan, Mandibahauddin, Phalia, Chhohranwala	32°31′39.89″N, 73°44′0.63″E
***Meloidogyne arenaria***						
	T311	A3	N1	unknown (extracted from soil)	Italie, Monsampolo del Tronto, Marché	F9497-6
	T332	A2	N1	Solanaceae	France	E9085
	T393	A2	N1	*Echiocactus grusonii*	Netherlands, greenhouse	E9279
	T411	A2	N1	*Calathea*	Costa Rica	F0428
	M41	A2	N1	*Allium cepa*	Tanzania, Morogoro, Msufini	6°17′15.432″S, 37°28′38.675″E
	T453	A2	N3	*Livistonia*	Sri Lanka	E9211
	T461	A2	N3	*Hosta*	USA	C8526
	Y19	A2	N3	*Dioscorea* (Yam)	Nigeria, Benue, Otukpo	7°11′31″N, 8°7′59″E
	Y34	A2	N3	*Dioscorea* (Yam)	Nigeria, Niger, Tufakampani	9°14′29″N, 6°54′59″E
***Meloidogyne*****sp. 1**						
	T473	A2-S1-M1	N1	*Heliconia*	Tanzania	E8465
	T585	A2-S1-M1	N1	*Ficus*	China	D2055-1
***Meloidogyne*****sp. 2**						
	T316	A1a-S1	N1	*Beta vulgaris*	Spain	C7720
	T576	A1a-S1	N1	*Solanum lycopersicum*	Guatemala	C7729
***Meloidogyne luci***						
	T326	L3	N1	*Solanum lycopersicum*	Dornberg, Slovenia	D9742
	T459	L3	N1	*Solanum lycopersicum*	Guatemala	F0034
	T693	L3	N1	*Daucus carota*	Iran	E4271
***Meloidogyne inornata***						
	T638	I3	N1	*Solanum lycopersicum*	Chili	F2484
	T695	I3	N1	*Solanum lycopersicum*	Chili	F2642
***Meloidogyne ethiopica***						
	T612	E3	N1	*Solanum lycopersicum*	Brazil, Charchar, received from R. Carneiro	E6089

Additional information on sampling location and origin of the studied material. If samples were collected from the field, GPS coordinates are provided, if the studied material originated from a reference culture the unique identification code is provided (National Plant Protection Organization, Wageningen).

**Table 2:** Sequences of newly developed primers and the position of the amplified fragment in relation to the total length of the mitochondrial coding sequence.

Gene (Length of CDS in bp^a^)	Primer name	Primer sequence 5′–3′	Primer position^a^	Fragment length (bp)	Gene coverage (%)
Cytochrome c oxidase subunit 1 (1522)	COX1F	ATCCTCCTTTGATGATTGATGG	374	996	65
	COX1R	AACTCAATAAAGAACCAATAGAAG	1369		
Cytochrome c oxidase subunit 2 (693)	COX2F	TTGAATTTAAGTGTTGTTTATTAC	155	432	62
	COX2R	GATTAATACCACAAATCTCTGAAC	586		
Cytochrome c oxidase subunit 3 (762)	COX3F	TTTTGCTGAGGATTAATAGG	171	397	52
	COX3R	TAAACTTCCATAAATACCATCAC	567		
NADH dehydrogenase subunit 1 (850)	NAD1F2	ATTAGATTATTAACTTTACTGGAGCG	40	558	66
	NAD1R2	GGAAAGAGAAAGTGAATTAGTGAGA	597		
NADH dehydrogenase subunit 2 (802)	NAD2F	GTATTATTAATATTTTGTAGGAAT	103	610	76
	NAD2R	ATATTAACTGACTTATTATCCC	712		
NADH dehydrogenase subunit 3 (315)	NAD3F	AATGAAAAATTCTTATTTCGAAAG	75	219	70
	NAD3R	ATATATTTTCATTCCAAAACTAAA	293		
NADH dehydrogenase subunit 5 (1474)	NAD5F2	TATTTTTTGTTTGAGATATATTAG	257	610	41
	NAD5R1	CGTGAATCTTGATTTTCCATTTTT	866		
Cytochrome b (1015)	CYTBF	TGAGGTTAATAATGGTTGGTTAATTCG	165	801	79
	CYTBR	GGGAGCCAAGAACCAGTTTT	965		
16S ribosomal RNA (804)	16SF	GCTCATTGTTAAAGAAAAGC	339	399	50
	16SR	GTTGTGAAATAGAGTTGTT	737		

^a^Length of the coding sequence and primer position within the gene are given according to mitochondrial genome of *Meloidogyne incognita*^46^.
