# Supplementary material for: Mitochondrial coding genome analysis of tropical root-knot nematodes (Meloidogyne) supports haplotype based diagnostics and reveals evidence of recent reticulate evolution
Source: Sci Rep. 2016 Mar 4;6:22591. doi: 10.1038/srep22591 (PMC4778069; doi:10.1038/srep22591)
Supplement: Supplementary Information [file srep22591-s1.pdf]

Supplementary information to

Mitochondrial coding genome analysis of tropical root-knot nematodes (*Meloidogyne*) supports haplotype based diagnostics and reveals evidence of recent reticulate evolution

Toon Janssen [1,*], Gerrit Karssen [1,2], Myrtle Verhaeven [1], Danny Coyne [3], Wim Bert [1]

[1] Nematology Research Unit, Department of Biology, Ghent University, K.L. Ledeganckstraat 35, 9000 Ghent, Belgium.
[2] National Plant Protection Organization, Wageningen Nematode Collection, P.O. Box 9102, 6700 HC Wageningen, The Netherlands.
[3] International Institute of Tropical Agriculture (IITA), c/o icipe, Kasarani, P.O. Box 30772-00100, Nairobi, Kenya.
[*]toon.janssen@ugent.be

16S

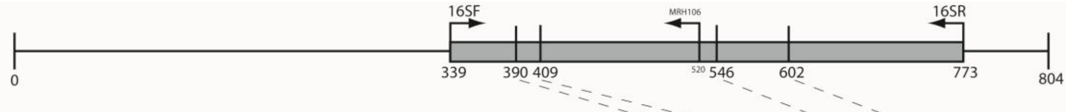

		390	409	546	602
Position within gene		390	409	546	602
Position within amplified region		52	71	208	264
Lineage	# populations				
M. incognita	12	A	G	A	G
M. javanica	9	G	A	A	A
M. sp. 2	2	G	G	G	G
M. luci	2	G	G	A	G
M. arenaria	7	G	G	A	G
M. ethiopica	1	G	G	A	G
M. sp. 1	2	G	G	A	G
M. inornata	2	G	G	A	G
M. floridensis	1	G	G	A	G

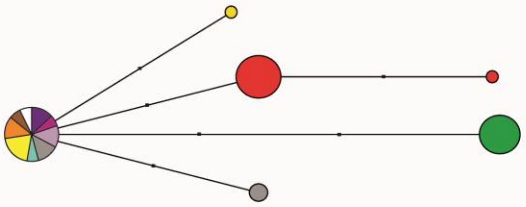

Cox2

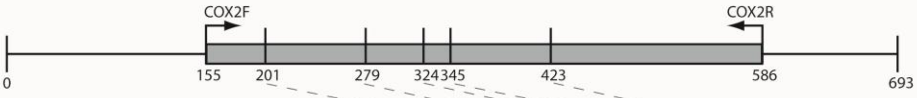

Position within gene		201	279	324	345	423
Position within amplified region		47	125	170	191	269
Lineage	# populations					
M. incognita	10	G	G	A	G	A
M. ethiopica	1	G	G	A	G	A
M. sp. 2	2	G	A	A	A	G
M. luci	3	A	A	T	G	A
M. sp. 1 H2	1	A	A	A	G	A
M. inornata	2	A	A	T	G	A
M. javanica	9	G	A	A	G	A
M. arenaria	8	G	A	A	G	A
M. sp. 1 H1	1	G	A	A	G	A
M. floridensis	1	?	A	A	G	A

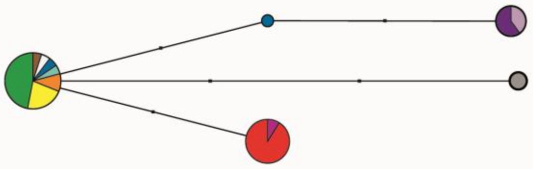

Cox3

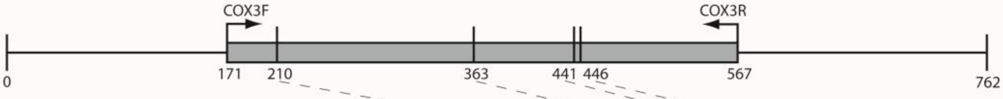

Position within gene		210	363	441	446
Position within amplified region		40	193	271	276
Lineage	# populations				
M. javanica	4	A	T	G	T
M. incognita	6	A	T	G	T
M. sp. 2	1	A	C	G	A
M. luci	3	G	T	G	T
M. arenaria	5	A	T	A	T
M. arenaria H2	2	A	T	G	T
M. ethiopica	1	G	T	G	T
M. sp. 1	2	A	T	G	T
M. inornata	1	G	T	G	T
M. floridensis	1	A	T	G	T

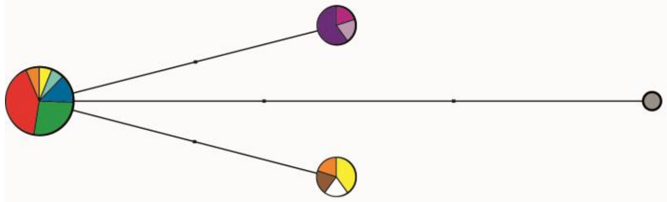

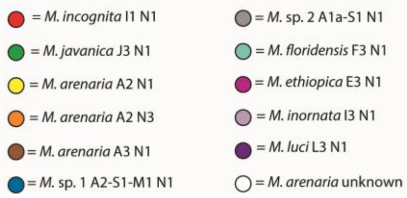

Figure S1. 16S, Cox2 and Cox3 gene sequence comparison between MIG lineages. For each gene fragment a schematic overview is provided, alongside an overview table and a haplotype network. The schematic overview shows the position and length of the amplified fragment, primer position and position of polymorphic nucleotide positions. The overview table shows the polymorphic nucleotide positions as well as the number of populations studied. The haplotype network shows the relationships between different haplotypes, circle size is equivalent to the number of studied populations and branch length is equivalent to the number of mutations (shown as black squares). Different isozyme phenotypes are displayed by different colours. Within the 16S gene two populations (*M. incognita* T532 and *M. arenaria* T332) have an extra mutation which are not shown in the schematic overview.

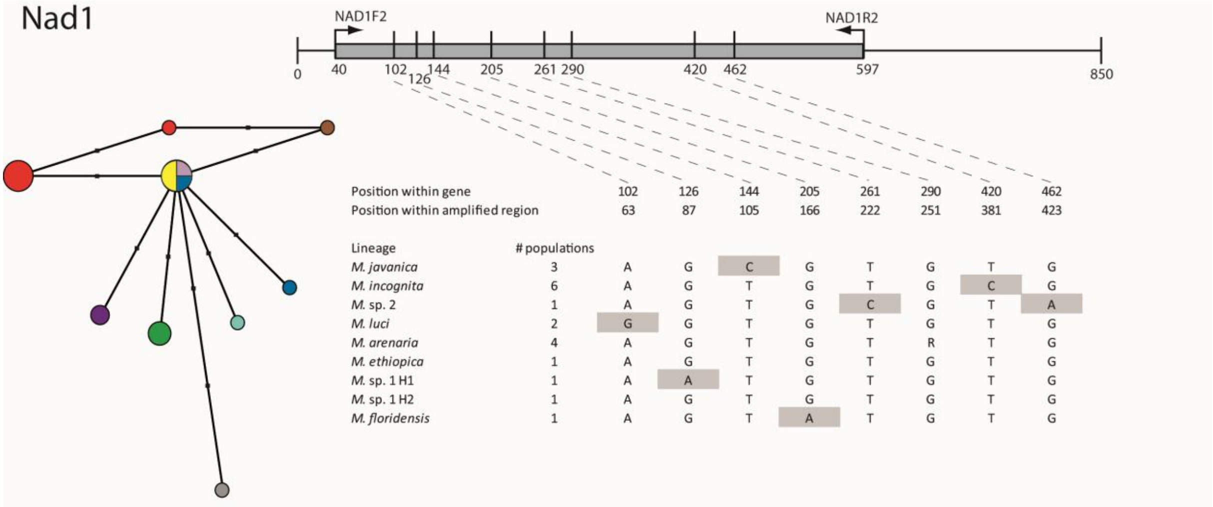

Position within gene		102	126	144	205	261	290	420	462
Position within amplified region		63	87	105	166	222	251	381	423
Lineage	# populations								
M. javanica	3	A	G	C	G	T	G	T	G
M. incognita	6	A	G	T	G	T	G	C	G
M. sp. 2	1	A	G	T	G	C	G	T	A
M. luci	2	G	G	T	G	T	G	T	G
M. arenaria	4	A	G	T	G	T	R	T	G
M. ethiopica	1	A	G	T	G	T	G	T	G
M. sp. 1 H1	1	A	A	T	G	T	G	T	G
M. sp. 1 H2	1	A	G	T	G	T	G	T	G
M. floridensis	1	A	G	T	A	T	G	T	G

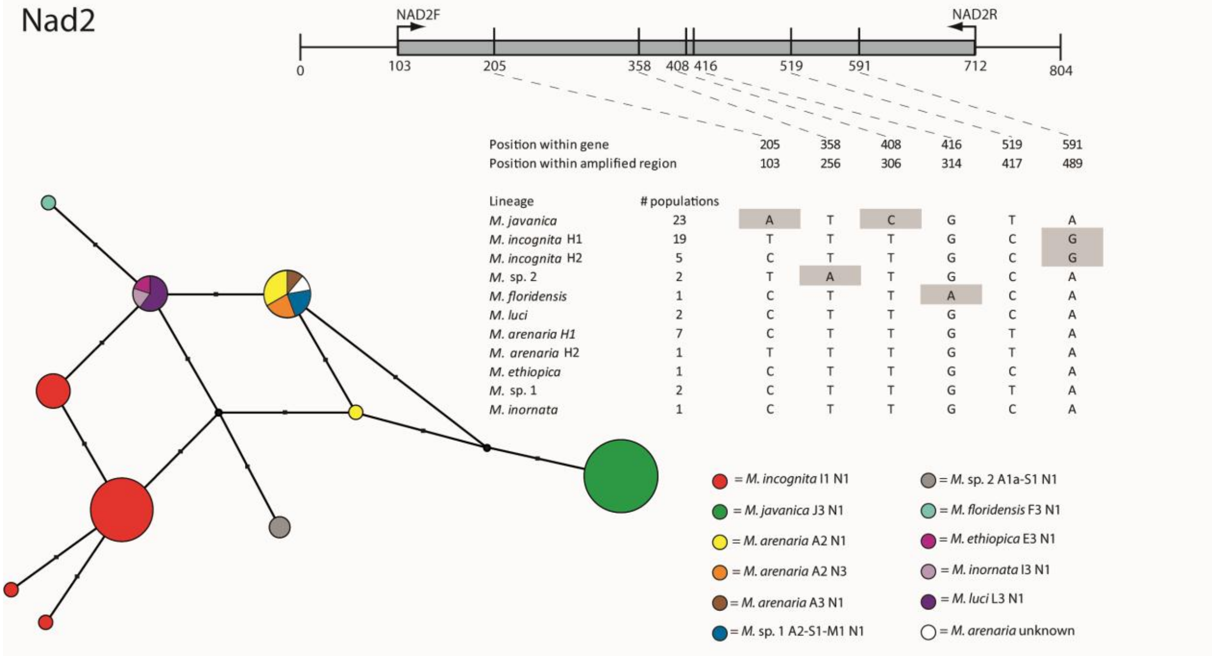

Position within gene		205	358	408	416	519	591
Position within amplified region		103	256	306	314	417	489
Lineage	# populations						
M. javanica	23	A	T	C	G	T	A
M. incognita H1	19	T	T	T	G	C	G
M. incognita H2	5	C	T	T	G	C	G
M. sp. 2	2	T	A	T	G	C	A
M. floridensis	1	C	T	T	A	C	A
M. luci	2	C	T	T	G	C	A
M. arenaria H1	7	C	T	T	G	T	A
M. arenaria H2	1	T	T	T	G	T	A
M. ethiopica	1	C	T	T	G	C	A
M. sp. 1	2	C	T	T	G	T	A
M. inornata	1	C	T	T	G	C	A

Cox1

Position within gene		558	603	846	969	1068	1119	1188
Position within amplified region		185	230	473	596	695	746	815
Lineage	# populations							
M. javanica	3	G	T	T	T	T	T	T
M. incognita	6	G	T	T	T	T	T	T
M. sp. 2	1	A	C	C	C	T	T	C
M. arenaria	4	G	T	T	T	T	T	T
M. ethiopica	1	G	T	T	T	T	T	T
M. sp. 1 H1	1	G	T	T	T	T	T	T
M. sp. 1 H2	1	G	T	T	T	T	C	T
M. inornata	2	G	T	T	T	T	T	T
M. floridensis	1	G	T	T	T	T	T	T
M. luci H1	2	G	T	T	T	C	T	T
M. luci H2	1	G	T	T	T	T	T	?

Cyt b

Position within gene		219	292	405	521	676	820	841
Position within amplified		55	128	241	357	512	656	677
Lineage	# populations							
M. javanica	3	A	A	A	C	T	G	G
M. incognita	6	A	A	A	C	T	G	G
M. sp. 2	1	G	A	A	C	T	G	G
M. luci	2	A	A	A	C	T	G	G
M. arenaria H1	1	A	A	A	C	T	G	A
M. arenaria H2	2	A	A	C/A	C	T	G	A
M. arenaria H3	1	A	G	A	C	T	G	G
M. ethiopica	1	A	A	A	C	A	G	G
M. sp. 1 H1	2	A	A	A	C	T	A	G
M. sp. 1 H2	2	A	A	A	C	T	G	G
M. floridensis	1	A	A	A	T	T	G	G

Figure S2. Nad1 and Nad2 gene sequence comparison between MIG lineages. For each gene fragment a schematic overview is provided, alongside an overview table and a haplotype network. The schematic overview shows the position and length of the amplified fragment, primer position and position of polymorphic nucleotide positions. The overview table shows the polymorphic nucleotide positions as well as the number of populations studied. The haplotype network shows the relationships between different haplotypes, circle size is

equivalent to the number of studied populations and branch length is equivalent to the number of mutations (shown as black squares). Different isozyme phenotypes are displayed by different colours, median vectors are shown as black circles. Within the Nad2 gene two *M. incognita* (C87 and M15) populations each have an extra mutation which are not shown in the schematic overview. For the Cox1 and Cytb fragment an overview table of the polymorphic nucleotide positions is provided.

Table S3: Overview of mitochondrial gene sequences used in this study with their respective Genbank accession numbers

Species	Specimen ID	16S	Cox1	Cox2	Cox3	Cytb	Nad1	Nad2	Nad3	Nad5
Meloidogyne enterolobii										
	T337	KU372433	KU372161	KU372188	KU372222	KU372243	KU372260		KU372335	KU372359
	T382			KU372186						
	T424									
	T441	KU372435		KU372187						
	T463	KU372431		KU372189						KU372358
	T468	KU372434		KU372184	KU372223	KU372242	KU372259		KU372336	
	T536	KU372432		KU372185						
	(1)	KP202351	KP202351	KP202351	KP202351	KP202351	KP202351	KP202351	KP202351	KP202351
Meloidogyne incognita										
	T384	KU372444	KU372165		KU372225			KU372284	KU372338	KU372373
	T161	KU372438		KU372198			KU372263	KU372288		KU372388
	T515	KU372442*		KU372195*				KU372289*		KU372372*
	T526	KU372445		KU372191				KU372299		KU372361
	T532	KU372437*		KU372193*				KU372292*		KU372386*
	T540	KU372443	KU372164	KU372196	KU372228	KU372246	KU372265	KU372287	KU372339	KU372366
	T552	KU372446		KU372197				KU372285		KU372381
	Y29									KU372362
	Y57									KU372374
	C33									KU372365
	C41	KU372440				KU372248		KU372293		KU372382
	C49							KU372282		KU372379
	C53									KU372364
	C69							KU372297		KU372368
	C81							KU372296		KU372369
	C87							KU372281		KU372376
	C95							KU372298		KU372370
	M4							KU372294		KU372363
	M8							KU372300		KU372380
	M15							KU372286		KU372371
	M20							KU372283		KU372383
	M21							KU372291		KU372384
	M28	KU372439	KU372163	KU372194	KU372226	KU372247	KU372264	KU372302	KU372340	KU372377
	M44							KU372301		KU372387
	M46									KU372375
	M49									KU372367
	A1	KU372441	KU372166	KU372192	KU372227	KU372245	KU372262	KU372290	KU372341	KU372378
	A3							KU372295		KU372385
	(2)	KJ476151	KJ476151	KJ476151	KJ476151	KJ476151	KJ476151	KJ476151	KJ476151	KJ476151
	(3)	CABB00000	CABB000	CABB00000	CABB000	CABB000	CABB000	CABB00000	CABB000	CABB00000

	000	00000	000	00000	00000	00000	000	00000	000
Meloidogyne javanica									
T347	KU372450	KU372169	KU372208	KU372231	KU372249	KU372266	KU372319	KU372343	KU372392
T417	KU372451		KU372204				KU372318		KU372391
T429	KU372453*		KU372206*				KU372321*		KU372401*
T485	KU372454		KU372201				KU372311		KU372410
T497	KU372455		KU372207				KU372320		KU372394
T509	KU372448		KU372205						KU372398
T520	KU372452		KU372203				KU372317		KU372396
Y60									KU372416
C35							KU372312		KU372415
C47							KU372313		KU372413
C63							KU372325		KU372407
C89							KU372324		KU372412
M14							KU372314		KU372397
M30							KU372305		KU372408
M39							KU372315		KU372409
M40				KU372230			KU372310	KU372344	KU372405
M50									KU372395
A8							KU372308		KU372393
A21							KU372316		KU372414
A23							KU372306		KU372399
A24							KU372322		KU372406
A25							KU372304		KU372402
A29							KU372309		KU372404
A30							KU372323		KU372400
A31									KU372411
A32	KU372449	KU372170	KU372202	KU372232	KU372250	KU372267	KU372307	KU372345	KU372403
(1)	KP202352	KP202352	KP202352	KP202352	KP202352	KP202352	KP202352	KP202352	KP202352
Meloidogyne arenaria									
T311	KU372428	KU372159	KU372177	KU372217	KU372240	KU372256	KU372278	KU372333	KU372357
T332	KU372426*	KU372160	KU372181*	KU372219	KU372239	KU372258	KU372277*	KU372334	KU372354*
T393	KU372430*		KU372183*	KU372221			KU372279*		KU372350*
T411	KU372425		KU372178				KU372274		KU372356
M41	KU372424	KU372158	KU372179	KU372220	KU372241	KU372257	KU372273	KU372332	KU372349
T453	KU372429		KU372180	KU372218			KU372276		KU372351
T461	KU372427		KU372182	KU372216			KU372275		KU372352*
Y19									KU372355
Y34									KU372353
(1)	KP202350	KP202350	KP202350	KP202350	KP202350	KP202350	KP202350	KP202350	KP202350
***Meloidogyne* sp. 1**									
T473	KU372458	KU372174	KU372212	KU372236	KU372253	KU372271	KU372329	KU372342	KU372420
T585	KU372459	KU372175	KU372213	KU372237	KU372254	KU372270	KU372328	KU372347	KU372421*
***Meloidogyne* sp. 2**									
T316	KU372460	KU372176	KU372214	KU372238	KU372255	KU372272	KU372331	KU372348	KU372423*
T576	KU372461		KU372215				KU372330		KU372422
Meloidogyne luci									
T326	KU372457	KU372173	KU372210	KU372233	KU372252	KU372268	KU372327	KU372346	KU372417*
T459	KU372456	KU372171	KU372211	KU372235	KU372251	KU372269	KU372326		KU372418

T693		KU372172	KU372209	KU372234					KU372419*
Meloidogyne inornata									
T638	KU372447	KU372167	KU372200				KU372303		KU372390
T695		KU372168	KU372199	KU372229					KU372389
Meloidogyne ethiopica									
T612	KU372436	KU372162	KU372190	KU372224	KU372244	KU372261	KU372280	KU372337	KU372360*
Meloidogyne floridensis									
(4)	CCDZ00000000	CCDZ00000000	CCDZ00000000	CCDZ00000000	CCDZ00000000	CCDZ00000000	CCDZ00000000	CCDZ00000000	CCDZ00000000

(1) Mitochondrial coding genes extracted from complete mitochondrial genome sequence [1]
(2) Mitochondrial coding genes extracted from complete mitochondrial genome sequence [2]
(3) Mitochondrial coding genes extracted from next generation sequencing reads of complete genome [3]
(4) Mitochondrial coding genes extracted from next generation sequencing reads of complete genome [4]
*This gene was sequenced for two different individuals from the same population, revealing the same haplotype

References

1 Humphreys-Pereira, D. A. & Elling, A. A. Mitochondrial genome plasticity among species of the nematode genus *Meloidogyne* (Nematoda: Tylenchina). *Gene* **560**, 173-183, doi:10.1016/j.gene.2015.01.065 (2015).

2 Humphreys-Pereira, D. A. & Elling, A. A. Mitochondrial genomes of *Meloidogyne chitwoodi* and *M. incognita* (Nematoda: Tylenchina): Comparative analysis, gene order and phylogenetic relationships with other nematodes. *Mol Biochem Parasit* **194**, 20-32, doi:10.1016/j.molbiopara.2014.04.003 (2014).

3 Abad, P. *et al.* Genome sequence of the metazoan plant-parasitic nematode *Meloidogyne incognita*. *Nat Biotechnol* **26**, 909-915, doi:10.1038/Nbt.1482 (2008).

4 Lunt, D. H., Kumar, S., Koutsovoulos, G. & Blaxter, M. L. The complex hybrid origins of the root knot nematodes revealed through comparative genomics. *Peerj* **2**, doi:10.7717/peerj.356 (2014).
